# 22Q11.2 deletion syndrome and psychosis: About a case

**DOI:** 10.1192/j.eurpsy.2021.1669

**Published:** 2021-08-13

**Authors:** C. García-Bernal, O. Neth, M. Ruiz Veguilla, N. Garrido Torres

**Affiliations:** 1 Mental Health, Virgen del Rocío Universitary Hospital, Spain, Spain; 2 Paediatrics, Virgen del Rocío Universitary Hospital, Spain, Spain

**Keywords:** immunodeficiency, psychosis, neurodevelopment

## Abstract

**Introduction:**

We present the case of a boy born in 2002 who was diagnosed with 22q11.2 DS at the age of 2 years. He was referred to neurology at age 9 for “attention deficits and irritability.” At age 12 he was referred to mental health for “irritability and anxious and depressive symptoms.” The boy was erroneously discharged with a diagnosis of “only” emotional disorder without subsequent follow-up. The evolution of this case resembles the evolution of others already described in the literature.

**Objectives:**

To demonstrate the lack of knowledge of the variety of comorbid disorders in this syndrome (20 to 40% present psychotic symptoms).

**Methods:**

Bibliographic search in the Pubmed database.

**Results:**

There is a partial T-cell immunodeficiency in 22q11.2DS patients confirmed by significantly reduced percentages of circulating T and helper T cells. An increased percentage of Th17 was found in adults with psychotic symptoms compared to non-psychotic adults in one article. The percentage of Th17 was related to the presence of positive psychotic symptoms. Another study says higher levels of IL-17 were found in patients with fewer symptoms. The importance of Th17 and IL-17 in the development of the hippocampus and of Th17 in the development of psychosis is highlighted. In those patients, there is a high IL-6 / IL-10 ratio in favor of a pro-inflammatory state. High levels of IL-6 are correlated with greater neurocognitive deficits and negative symptoms.

**Conclusions:**

1. There is evidence for a theory of inflammation in psychosis development. 2. The 22q11.2 DS could be used as a research model.

**Disclosure:**

No significant relationships.

